# Examining developmental assets of young Black sexual gender minority males in preventing suicidal behaviors

**DOI:** 10.1016/j.jpsychires.2024.01.035

**Published:** 2024-01-25

**Authors:** Donte T. Boyd, Kristian V. Jones, David J. Hawthorne, Camille R. Quinn, Amelia C. Mueller-Williams, S. Raquel Ramos, Typhanye V. Dyer, Leo Wilton

**Affiliations:** a College of Social Work, The Ohio State University, Columbus, OH, USA; b School of Social Work, University of Washington, USA; c Department of Behavioral and Community Health, University of Maryland, College Park, MD, USA; d Center for Equitable Family & Community Well-Being, School of Social Work, University of Michigan, Ann Arbor, MI, USA; e Addiction Center, Department of Psychiatry, School of Medicine, University of Michigan, MI, USA; f School of Nursing and School of Public Health, Yale University, New Haven, CT, USA; g Department of Epidemiology and Biostatistics, University of Maryland, College Park, MD, USA; h Department of Human Development, State University of New York at Binghamton, Binghamton, NY, USA; i University of Johannesburg, Department of Humanities, South Africa

**Keywords:** Black men who have sex with men, Suicide, Depression symptoms, Developmental assets

## Abstract

Black gay and bisexual male adolescents and young adults (BGBMA/YA) are at higher risk for suicidal outcomes given their minoritized and stigmatized identities at the intersection of race and sexual orientation. This study explores key developmental assets, including family support and family communication, and their role in preventing depression symptoms and suicidal outcomes among BGBMA/YA. A cross-sectional survey was administered to participants (N = 400, *M*_*age*_ = 23.46, SD = 2.59) recruited through Amazon M-Turk, community-based organizations, and social media sites. A path analysis was conducted to examine associations among external assets (family support, communication about sex and drugs with parents, open family communication), depression symptoms, and suicidal attempts and plan to die by suicide. About 28 % of respondents reported a suicide attempt in the past 12 months. Depression symptoms and communication about sex and drugs with parents were positively associated with plan to die by suicide. Family support was negatively associated with depression symptoms. Depression symptoms were positively associated with suicide attempts. Family support was indirectly and negatively associated with suicide attempts. Suggestions for future research and policy implications are discussed.

## 1. Introduction

Suicide rates for both youth and young adults in the United States have increased over the past few decades, especially among those identifying as Black (CDC, 2021; Miron et al., 2019; Xu et al., 2020; Wang et al., 2020). According to the CDC, suicide rates among Black Americans peak during the period of adolescence and young adulthood and then decrease for the remainder of their lives (CDC, 2021). From 2010 to 2020, Black male adolescents and young adults (ages 15–24) in the United States recorded a suicide rate four times greater than the rate for Black female adolescents and young adults (CDC, 2021). Further, research also suggests that sexual orientation is associated with a higher risk of suicide attempts in young people who identify as gay or bisexual (Baams et al., 2015; King et al., 2008; Miranda-Mendizábal et al., 2017; Stone et al., 2014). Black gay and bisexual male adolescents and young adults (BGBMA/YA) also have elevated risk for suicidal outcomes (i.e., suicidal ideation, suicide attempts, or completed suicides (Boyd et al., 2021; Goodwill et al., 2021; Meyer, 2003; Silenzio et al., 2007; Wilton et al., 2018). Despite the significance of the intersection of the adolescent developmental period and identifying as LGBTQ, not much understanding exists on factors that could mitigate suicidal thoughts and outcomes among BGBMA/YA. Hence, this study is one of few to examine the protective factors that could buffer suicidal outcomes for this specific population of young people (Boyd et al., 2021).

### 1.1. Suicidal behaviors and risk factors among male gay and bisexual adolescents and young adults

Adolescence is a crucial stage of life for gay and bisexual males because of the unique environmental and social factors such as social stress and lack of social networks (Dodge et al., 2012) that can impact mental health outcomes for this specific demographic (Haas et al., 2010; Hatchel et al., 2021; Plöderl and Tremblay, 2015; Russell and Toomey, 2012). During this developmental stage, factors such as family rejection, anti-lesbian/gay/bisexual stigma, school victimization, bullying, and internalized homophobia have been associated with elevated risk for negative psychosocial and emotional sequelae, including a higher risk of suicidal behaviors for gay and/or bisexual youth and young adults (Fish et al., 2019; Johnson Leibowitz et al., 2019; Kelleher, 2009; Kosciw et al., 2016; Russell et al., 2011). Due to the myriad of challenges gay and bisexual youth and young adult males experience, research in this area has consistently highlighted the importance of protective factors and external assets (e.g., interpersonal factors) that can buffer the negative effects of exposure to these challenges, on mental health (e.g., depressive symptoms) and suicidality (Russell and Fish, 2016).

### 1.2. Depression and suicidal outcomes

Extant literature has highlighted that sexual and gender minority youth and young adults are at higher risk for symptoms of depression, and that these symptoms are associated with suicidal outcomes in this population (Baams et al., 2015; Goodwill et al., 2021; Hatzenbuehler, 2009; Johnson Leibowitz et al., 2019; Kosciw et al., 2012; Martin-Storey and Crosnoe, 2012; Mustanski and Liu, 2013; Plöderl and Tremblay, 2015). The increased risk for depression in this group can be attributed to several factors including discrimination, victimization, and stigma (Johnson Leibowitz et al., 2019). Correspondingly, external assets such as family acceptance and family connectedness have been identified as protective factors that act to mitigate depression and suicidal outcomes (Ryan et al., 2009, 2010). Although research has consistently identified family (e.g., parental rejection, family support) as an important resource in the lives of BGBMA/YA (Blosnich and Bossarte, 2012; Hatchel et al., 2021), there is limited empirical data examining how family factors can act as protective mechanisms for suicidal outcomes for BGBMA/YA.

### 1.3. Protective mechanisms and suicidal outcomes

There is a dearth of research on protective mechanisms that explain lower suicide risk among Black gay or bisexual males and all LGB youth (Toomey et al., 2019). However, the literature that does exist is promising. For instance, one study found that youth who were attracted to the same sex and had supportive teachers, and a strong sense of belonging were less likely to report suicide ideation (Russell and Toomey, 2012; Whitaker et al., 2016). In another study, positive social support among LGB youth was linked to lower distress (McConnell et al., 2016; Snapp et al., 2015), and an increase in parental support was associated with a lower risk for suicidal behaviors (Ryan et al., 2010). Much of the literature that concentrated on external support has not focused on Black gay and bisexual males and their family support.

### 1.4. Developmental Assets Framework

The Developmental Assets Framework identifies skills, experiences, relationships, and behaviors that build upon one another to create a healthy developmental stage for young people (Scales et al., 2006). The Search Institute identifies 40 youth developmental assets with 20 external (e.g., community, family support, social support, and neighborhood environment) and 20 internal assets (e.g., commitment to learning, positive values, social competencies, and positive identity) (Scales, 1999; Taliaferro et al., 2020). Research suggests that external assets contribute to reducing the risk of negative outcomes such as engaging in risky behaviors and depression (Zheng et al., 2022) for youth across all racial and ethnic groups, including those who identify as sexual and gender minorities (Johnson Leibowitz et al., 2019). However, protective mechanisms against suicide risk among sexual and gender minority youth are understudied (Toomey et al., 2019). The few existing studies show that high social support could contribute to lower distress in sexual and gender minority youth (McConnell et al., 2016; Snapp et al., 2015), and that parental support could help lower the risk of suicidal behavior (Ryan et al., 2010). This study is the first of its kind to examine how family constructs as an external asset might influence depression and suicidal behaviors among Black gay and bisexual men and help prevent suicidal outcomes.

This study addresses the gap in the literature on protective factors for suicidal behaviors among Black men who have sex with men (MSM) ages 18 to 29 guided by the Developmental Assets Framework (DAF). We hypothesized: (1) family support will be directly associated with depression symptoms and suicidal planning; (2) open family communication will be directly associated with depression symptoms and suicidal planning; (3) communication about sex and drugs with parents will be directly associated with both depression symptoms and suicidal planning; (4) family support will be indirectly associated with suicide attempts; (5) Open family communication will be indirectly associated with suicide attempts; and (6) communication about sex and drugs with parents will be indirectly associated with suicide attempts.

## 2. Methods

### 2.1. Study procedures and recruitment

This study used data from a larger study that examined strength-based approaches to sexual, physical, and mental health and suicidal behaviors among young Black men aged 18 to 29 who have sex with men (Boyd et al., 2023). The survey was programmed with Qualtrics software for different sampling sites. An anonymous link was generated and included on a recruitment flyer. The flyer was then distributed via social media sites (Facebook and Twitter) and was provided to community-based organizations and Amazon Mechanical Turk (MTurk) (Beymer et al., 2018). The principal investigator and research assistants distributed the survey via social media every morning at 8 a.m. Eastern Standard Time.

Mechanical Turk provides a cost-effective and rapid method of recruitment for research studies that span multiple disciplines, including public health (Pacek et al., 2017). To view and participate in the survey, individuals who were registered with MTurk were required to have a 95 % or higher approval rating from previous surveys, be age 18 or older, and reside in the United States, as confirmed during the initial MTurk registration (Carter et al., 2014; Pacek et al., 2017; Rass et al., 2015). In addition, potential respondents who logged on to the MTurk platform during the week in which the survey was administered were informed that they had an opportunity to take a “survey about strength-based approaches to sexual, physical, and mental health and suicidal behaviors among young Black men who have sex with men aged 18 to 29”. Participants were informed that the survey would take 20 min and that surveys were released every morning at 8 a.m. Eastern Standard Time. Participants were instructed to complete the survey in one sitting, were paid one dollar, and were given other incentives from MTurk (Pacek et al., 2017).

For community-based organizations, the research team shared the flyer with community health workers, and they distributed the flyer to eligible participants who were considered clients of their organization. Participants were recruited on December 1, 2021, and January 31, 2022. All individuals who completed the 20-min survey and provided an email address received a $35 electronic Amazon gift card.

To ensure data quality and reduction of bots, our survey used Qualtrics survey protection, and the research team checked IP addresses to ensure that respondents were in the United States and maintained data integrity by not allowing the same respondent to answer the eligibility or survey questions more than once. In addition, we used speeding checks (respondents with a survey duration of ≤ one-third of the median duration of the survey), and participants who failed this quality check were excluded from the final sample. Qualtrics survey protection allowed us to use tools to prevent ballot box stuffing (a tool that places a cookie in the browser once a person has submitted a response), reCAPTCHA (Completely Automated Public Turing Test to tell Computers and Humans Apart) scores (a question placed before the survey asking respondents to identify certain items in pictures or replicate a series of letters), and bot detection (a Qualtrics survey question that indicates a reCAPTCHA score that relates to the probability that the respondent is a bot).

Upon clicking on the survey link, participants were provided an informed consent form and asked to complete a screening tool to assess their eligibility for the study. Individuals who met the inclusion criteria were asked a series of questions on demographics; strength-based assets such as family support and communication; sexual, physical, and mental health, and suicide behaviors. Participants who used social media sites and MTurk to complete the survey used their personal computers. Individuals who completed the survey in a community-based organization used a computer or tablet provided by the organization. The study was approved by the Ohio State University Institutional Review Board (IRB # 2021E1175).

### 2.2. Participants

The inclusion/exclusion criteria were the same for all sampling sites. Respondents were eligible to participate in the study if they self-identified as Black or African American, were aged 18 to 29, resided in the United States, were assigned as male at birth, were fluent in English, currently identified as a man, and reported sexual contact (oral, anal, or otherwise) with a male in the previous year. Ineligible respondents were exited from the survey immediately upon providing a response that did not meet the inclusion criteria. We used the Qualtrics forced response option to ensure every participant answered each question.

We had a final sample of 400 Black gay and bisexual males ages 18 to 29 (*M* = 23.46; *SD* = 2.59). The majority of participants (*n* = 200 were recruited from MTurk), followed by community-based organizations (*n* = 100), and social media sites (*n* = 100). The majority of the sample identified as Black American or African American (75 %), followed by Caribbean (10 %), Afro-Latino (10 %), and 5 % self-identified as continental African. Twenty-eight percent of the sample had never attended high school and 29 % had completed college or postgraduate studies. According to the United States Census, 39 % of 18- to 24-year-old, are enrolled in college, and among those above 25, 22.6 % have graduated with a college degree or higher. This sample reported higher numbers of youth and young adults having degrees.

The average household income ranged from less than $20,000 to $150,000, with the average household income being $58,000. Most of the sample (95 %) reported being assigned male at birth and 5 % as female. 100 % self-reported having sex with men within the last year. Forty-five percent of the sample reported being gay, 35 % reported being straight or heterosexual, 10 % bisexual, 5 % questioning, and 5 % other.

### 2.3. Measures

#### 2.3.1. Outcome variable

##### Past year suicide attempt.

Attempting suicide was measured using a single item that asked respondents to indicate whether they had attempted to end their life within the past 12 months. Response categories were (1 = yes, 0 = no) (Goodwill et al., 2021).

#### 2.3.2. Mediator variables

##### Depression Symptoms.

We used the Center for Epidemiological Studies Depression Scale (CESD-10) to measure depression symptoms. The CESD-10 assesses depression symptoms experienced in the past week. It has been validated among clinically depressed populations, the general population, and sexual minorities of color (English et al., 2020). Sample items include “How many times in the past week did you feel as good as other people?” and “How many times in the past week did you have trouble keeping your mind on task?” Response options range from 0 (rarely or never) to 3 (most or all of the time). The CESD-10 scores range from 0 to 30, with higher scores indicating more depression symptoms (Cronbach alpha = .81). Individuals who had scores above 20 were classified as having moderate-to-severe depression symptoms (Andresen et al., 1994; Okafor et al., 2020). Any score above 10 means the young men are depressed.

##### Past year suicide plan.

‘Planned to die by suicide’ was measured using a single item that asked participants whether they had made a plan to end their life within the past 12 months. Response categories were 1 (yes) or 0 (no) (Goodwill et al., 2021).

#### 2.3.3. Independent variables

##### External Assets.

Family support was measured using 3 items with a 5-point Likert scale ranging from 1 (*strongly disagree*) to 5 (*strongly agree*). Participants were asked questions such as “My parents give me help and support when I need it.” We averaged the three items and higher scores indicate more family support, and the Cronbach alpha was α = .91 (Leffert et al., 1998; Scales, 1999; Syvertsen et al., 2021). Open family communication was measured using a single item on a 5-point Likert scale ranging from 1 (*strongly disagree*) to 5 (*strongly agree*). Participants were asked the following question “I have lots of good conversations with my parents.” (Leffert et al., 1998; Scales, 1999; Syvertsen et al., 2021). Communication about sex and drugs with parents was measured using a single item ranging from 1 (*never*) to 5 (*all of the time*) asking respondents the following: “If you had an important concern about drugs, alcohol, sex, or some other serious issue, would you talk to your parent(s) about it?” (Leffert et al., 1998; Scales, 1999; Syvertsen et al., 2021).

#### 2.3.4. Statistical analysis

Table 1 presents the descriptive statistics of the study sample, while Table 2 presents the bivariate correlations of key study variables that were conducted (see Table 2). We used Mplus Version 8.3 to conduct a path analysis to test our hypotheses examining whether external assets (family support, open family communication, and communication about sex and drugs with parents) were directly associated with depression symptoms and suicide planning. In addition, we looked at whether external assets were indirectly associated with suicide attempts through depression symptoms and suicide planning. A path analysis also allowed us to test the indirect effects of external assets on suicidal attempts. The mean-and-variance adjusted weighted least squares estimator was used instead of maximum likelihood estimation, as this estimator is preferred when the dependent variable is categorical and when the data are not distributed normally (Kline, 2023; Muthén and Muthén, 2019). The percentage of missing data was less than 5 %. Full information maximum likelihood was used for missing data (Muthén and Muthén, 2019). The goodness-of-fit was assessed with measures using the chi-square test, Akaike information criterion (AIC) and Bayes information criterion (BIC) because the dependent variable was categorical. In addition, standardized beta coefficients and p-values were included and used to examine associations among study variables.

## 3. Results

Table 1 provides the demographic characteristics of the sample. Twenty-seven percent of the sample reported attempting suicide and 33 % reported planned to die by suicide. The average score for depression for this sample was 14 (SD = 5.97). Correlation results (Table 2) showed that suicide planning was positively associated with suicide attempts (r = 0.064, p < .001). Depression symptoms were positively associated with suicide attempts (r = 0.40, p < .001) and suicide planning (r = 0.44, p < .001). Open family communication was positively associated with suicide attempts (r = 0.20, p < .001), suicide planning (r = 0.19, p < .010), depression symptoms (r = 0.19, p < .001), and family support (r = 0.18, p < .010). Lastly, communication about sex and drugs with parents was positively correlated with open communication (r = 0.79, p < .001).

### Path Analysis.

**Fig. 1.**
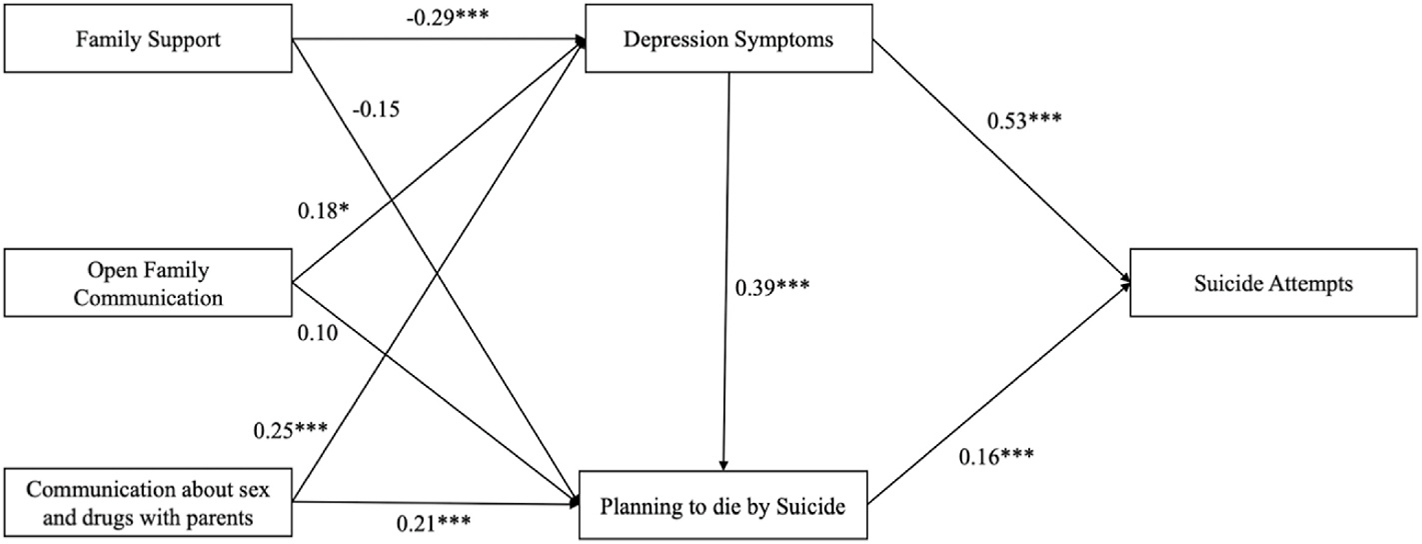
Path model testing direct effects from external assets to suicide attempts in the past year through depressive symptoms and planning to die by suicide in the past year (n = 400). Note: p < .05*; p < .01**, p < .001***; standardized betas reported.

**Fig. 2.**
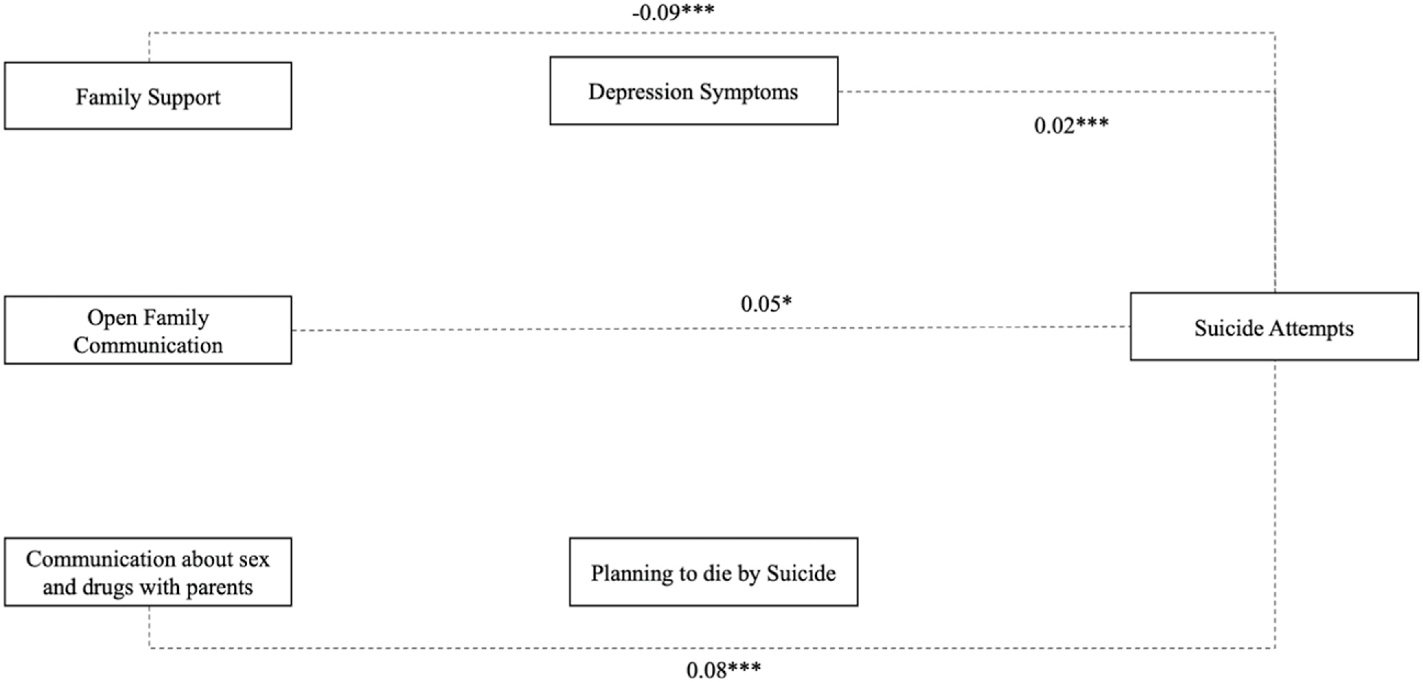
Path model testing indirect effects from external assets to suicide attempts in the past year through depressive symptoms and planning to die by suicide in the past year (n = 400). Note: p < .05*; p < .01**, p < .001***; unstandardized betas reported.

The path model representing the direct effects between external assets and suicide attempts is presented in Table 3 and Fig. 1. The model fit was assessed using the chi-square/df ratio = 10.168; *p* = .071, AIC = 4720.662 and BIC = 4809.792. Depression symptoms were directly and positively associated with young men who planned to die by suicide (β = 0.39, p < .001). Communication about sex and drugs with parents was directly and positively associated with a plan to die by suicide (β = 0.21, p < .001). Family support was directly and negatively associated with depression symptoms (β = −0.29, p < .001). Surprisingly, open family communication (β = 0.18, p = .043) and communication about sex and drugs (β = 0.25, p < .001) were both directly and positively associated with depression symptoms. Planning to die by suicide (β = 0.16, p < .001) and depression symptoms (β = 0.53, p < .001) were both directly and positively associated with suicide attempts. Our results indicated that family support was indirectly and negatively associated with suicide attempts (β = −0.09, p < .001). Both open family communication (β = 0.05, p < .001) and communication about sex and drugs with parents (β = 0.08, p < .001) were both positively and indirectly associated with suicide attempts, as illustrated in Table 4 and Fig. 2.

## 4. Discussion

This study was guided by the Developmental Assets Framework to explore how protective factors may contribute to the psychosocial wellbeing of BGBMA/YA. We examined how external assets (family support, communicating about sex and drugs with parents, and open family communication) influenced depression symptoms and suicidal behaviors in this population. Our hypotheses were partially supported, but in most cases not in the expected direction. Overall, external assets were positively associated with depression and suicide outcomes except for the indirect effect of family support on suicide attempts. While prior research has highlighted the benefits of external assets on a variety of health behaviors, including depression (Bleck and DeBate, 2016; Zheng et al., 2022), our findings extend the Developmental Assets framework to depression symptoms and suicide among BGBMA/YA, and suggest these factors may have a detrimental impact on wellbeing in this population.

Our results indicated family support lowered depression symptoms and reduced suicidality in this sample. Our findings are consistent with previous research that suggests family support serves as a protective mechanism against risk factors such as suicide and depression (McConnell et al., 2015; Quinn et al., 2022; Ryan et al., 2009a;2010b). This potentially means that BGBMA/YA believe that their parents are supportive when they need them, which reduces their depression and indirectly their suicidality (McConnell et al., 2015; Ryan et al., 2009a;2010b). Further, BGBMA/YA may feel their parents are loving and encouraging which could allow them to better communicate their challenges with depression and suicidality with their family (Boyd et al., 2022). These findings extend the literature on the importance of family support in the lives of BGBMA/YA and the influence that family has on their mental health and suicidality. Future research should continue to investigate the relationship between external assets and suicidality among BGBMA/YA. More research is needed to better understand the role of developmental assets as this is the first study to do so (Boyd et al., 2021; Toomey et al., 2019).

Our results suggested that communication about sex and drugs with parents and open family communication were both directly and indirectly and positively associated with both depression symptoms and suicidality among the study participants. These findings align with previous research that parent-youth communication may function differently in families with sexual gender minority youth especially after they disclose their sexual orientation (Guilamo-Ramos, et al., 2016). Previous literature also notes that lack of knowledge and high levels of discomfort between parents and SGM youth and young adults may lead to create negative discussions in the households and negative beliefs among SGM youth (McKay and Fontenot, 2020). These findings may suggest that the discomfort of the parents may lead to negative discussions about sex and drugs, and this communication might lead to the parents being seen as being judgmental and uncomfortable by their BGBMA/YA sons, which may result in depression and suicidality among them. Parents may need additional support on how to have honest discussions with their BGBMA sons about sex, drugs, and mental health. Lastly, future research could examine family dynamics and parenting roles to clearly understand how topics such as sex, drugs, and mental health are being communicated to BGBMA sons.

Consistent with previous research, we find a positive association between depression and suicide plans and attempts (Hatchel et al., 2019; Kaniuka et al., 2019). Notably, in this sample, 34 % reported making plans for suicide and 28 % reported a suicide attempt in the past year. These numbers are alarmingly higher than national estimates for lesbian, gay, and bisexual adults, with 12 % reporting suicide plans and about 6 % reporting a suicide attempt in the past 12 months (SAMHSA, 2020). Potential explanations for elevated suicidality among this sample are the elevated risks of victimization, trauma, and experiences of stigma experienced by BGBMA/YA (Mustanski and Liu, 2013). This community experiences multiple intersectional oppressions due to their identities as men who identify as gay or bisexual and Black, while also experiencing the heightened risk of depression and suicidality associated with adolescence and young adulthood (Turpin et al., 2019). It should also be noted that 83 % of respondents reported losing their jobs due to the COVID-19 pandemic. As such, elevated suicidality in this sample could also be due to the unprecedented and cumulative hardships posed by the pandemic (Banerjee and Sathyanarayana Rao, 2021).

### Limitations

Despite filling a significant gap in the literature, this study was not without limitations. This study has a cross-sectional design, and therefore cannot establish how external assets influence depression symptoms and suicidal behaviors over time. We also acknowledge that online data collection has its limitations, and we cannot for certain confirm that the sample included all BGBMA/YA. In addition, there are potential biases associated with self-reported data, including recall bias and social desirability, which may have influenced the responses to depression and suicide measures. It is possible that BGBMA/YA under-reported or over-reported depression and suicide measures. Also, the measurement items used may pose limitations in this study, as single items were used to measure suicidal behaviors, open family communication, and communication about sex and drugs. Future research could use some existing validated scales, as well as develop measures via exploratory sequential mixed methods studies to help further the research on external assets and suicide among this population to counter this constraint. Despite these limitations, the present study has significant implications for policy and practice with Black sexual minority young adults.

### Implications for practice and policy

There is a critical need to identify suicide risk reduction strategies that could also work to increase support for BGBMA/YA. Therefore, researchers and clinicians must consider the intersectionality of ways in which BGBMA/YA are marginalized to gain new perspectives on BGBMA/YA mental health and find innovative approaches to reduce risk and increase promotive factors. There must be an increase in funding support at the local, state, and federal levels that address the alarming rates of suicide for BGBMA/YA while also providing support that helps families navigate these often sensitive yet tough discussions on suicide and depression with their children.

### Future research and conclusion

The purpose of this study was to examine external assets on suicidal outcomes among BGBMA/YA. Our findings showed that positive family support lowered depression symptoms and indirectly suicide attempts. However, open family communication and communication about sex and drugs with parents increased depression symptoms and suicidality among BGBMA/YA, which was contrary to what was expected. BGBMA/YA may be able to have discussions with their family about a wide range of topics but might not feel comfortable engaging in conversation about depression and suicide. Future research should consider the types of open discussions that are happening between BGBMA/YA and their families to understand how this impacts their mental health. Moreover, future research should examine other external assets and internal assets such as positive identity and academic motivation to further explore how they impact BGBMA/YA and their mental health outcomes.

## Figures and Tables

**Table 1:** Demographics of study sample (N = 400).

Variable	M or %	SD or *N*	Range (if applicable)

Age	23.46	2.59	18–29
Household income	$57,499.50	1.34	Up to $150,000
Race and Ethnicity			
Black American or African American	75 %	300	
Caribbean (e.g., Jamaican, Haitian)	10 %	40	
Continental African (e.g., Nigerian, Ghanian)	5 %	20	
Afro-Latino (e.g., Dominican)	10 %	40	
Sex assigned at Birth			
Male	95 %	380	
Female	5 %	20	
Sexual Orientation			
Heterosexual or straight	35 %	140	
Gay	45 %	180	
Bisexual	10 %	40	
Questioning	5 %	20	
Other	5 %	20	
Education			
Never attended school	30 %	100	
Less than high school	19 %	65	
Some high school	4 %	12	
High school diploma or GED	2.4 %	8	
Some college, associate degree	14 %	47	
College, postgraduate	29 %	98	
Currently in school	1.5 %	5	
Laid off due to Covid 19			
Yes	83 %	250	
No, I was laid off for other reasons	14 %	50	
Not applicable, I was not working prior	14 %	50	
Depressive symptoms	14.46 %	5.97	
Family Support	5.17	1.52	1–5
Open family communication	3.88	1.03	1–5
Communication about sex and drugs with parents	3.52	1.22	1–5
Planning to die by suicide			
Yes	34 %	130	
No	66 %	220	
Suicide attempt			
Yes	28 %	98	
No	72 %	252	
No	69 %	224	

**Table 2:** Correlation of key study variables (n = 400).

Variable	1	2	3	4	5	6

Suicide attempts	1					
Plan to die by suicide	0.64***	1				
Depressive symptoms	0.40***	0.44***	1			
Family support	− 0.06	− 0.04	0.00	1		
Open family communication	0.20***	0.19**	0.19***	0.18**	1	
Communication about sex and drugs with parents	0.01	0.05	0.05	0.09	0.79***	1

* *p*<.05

** *p*<.01

*** *p*<.001.

**Table 3:** Direct Effects of External assets on Suicide Attempts through planned to die by suicide and depression symptoms (n = 400).

	B	β	SE	P-Value	95C%CI

Direct Effects					
Planned to die by suicide					
Depression symptoms	0.03	0.39***	0.04	0.001	0.29,0.48
Family support	− 0.02	− 0.15	0.08	0.08	− 0.31,0.02
Open family communication	0.02	0.10	0.08	0.25	−0.06 0.25
Communication about sex and drugs with parents	0.08	0.21***	0.06	0.001	0.08, 0.33
Depression symptoms					
Family support	− 0.65	0 29***	0.09	0.001	−0.46, −0.11
Open family communication	0.91	0.18*	0.08	0.043	−0.01, 0.35
Communication about sex and drugs with parents	1.30	0.25***	0.06	0.001	0.11, 0.38
Suicide attempts					
Planned to die by suicide	0.52	0.16***	0.05	0.001	0.06, 0.26
Depression symptoms	0.10	0.53***	0.04	0.001	0.45 0.62

Note:

* *p*<.05

** *p*<.01

*** *p*<.001

B= unstandardized beta; β = Standardized Betas; SE= standardized errors; CI: confidence intervals.

**Table 4:** Indirect Effects of External assets on Suicide Attempts through planned to die by suicide and depression symptoms (n = 400).

	B	SE	P-Value	95C%CI

Indirect Effects				
Suicide attempts				
Depression symptoms	0.02***	0.00	0.001	0.01,0.02
Family support	−0.09***	0.03	0.001	− 0.14, − 0.04
Open family communication	0.05*	0.02	0.035	
Communication about sex and drugs with parents	0.08***	0.02	0.001	0.04, 0.11

Note:

* *p* < .05

** *p* < .01

*** *p* < .001

B = unstandardized beta; SE = standardized errors; CI: confidence intervals.
